# Changes among US Cancer Survivors: Comparing Demographic, Diagnostic, and Health Care Findings from the 1992 and 2010 National Health Interview Surveys

**DOI:** 10.1155/2013/238017

**Published:** 2013-06-16

**Authors:** Natasha D. Buchanan, Jessica B. King, Juan L. Rodriguez, Arica White, Katrina F. Trivers, Laura P. Forsythe, Erin E. Kent, Julia H. Rowland, Susan A. Sabatino

**Affiliations:** ^1^Centers for Disease Control and Prevention, Division of Cancer Prevention and Control, Epidemiology and Applied Research Branch, 4770 Buford Highway N.E., MS K-55, Atlanta, GA 30341, USA; ^2^Centers for Disease Control and Prevention, Division of Cancer Prevention and Control, Cancer Surveillance Branch, Atlanta, GA 30341, USA; ^3^Cancer Prevention Fellowship Program, Division of Cancer Prevention & Office of Cancer Survivorship, National Cancer Institute, NIH/DHHS, Bethesda, MD 20892, USA; ^4^Patient Centered Outcomes Research Institute, Washington, DC 20036, USA; ^5^Office of Cancer Survivorship, Division of Cancer Control and Population Sciences, National Cancer Institute, NIH/DHH, Bethesda, MD 20892, USA

## Abstract

*Background*. Differences in healthcare and cancer treatment for cancer survivors in the United States (US) have not been routinely examined in nationally representative samples or studied before and after important Institute of Medicine (IOM) recommendations calling for higher quality care provision and attention to comprehensive cancer care for cancer survivors. *Methods*. To assess differences between survivor characteristics in 1992 and 2010, we conducted descriptive analyses of 1992 and 2010 National Health Interview Survey (NHIS) data. Our study sample consisted of 1018 self-reported cancer survivors from the 1992 NHIS and 1718 self-reported cancer survivors from the 2010 NHIS who completed the Cancer Control (CCS) and Cancer Epidemiology (CES) Supplements. *Results*. The prevalence of reported survivors increased from 1992 to 2010 (4.2% versus 6.3%). From 1992 to 2010, there was an increase in long-term cancer survivors and a drop in multiple malignancies, and surgery remained the most widely used treatment. Significantly fewer survivors (<10 years after diagnosis) were denied insurance coverage. Survivors continue to report low participation in counseling or support groups. *Conclusions*. As the prevalence of cancer survivors continues to grow, monitoring differences in survivor characteristics can be useful in evaluating the effects of policy recommendations and the quality of clinical care.

## 1. Introduction

Advances in cancer detection, diagnosis, and treatment, along with the aging of the United States (US) population, have resulted in a large and growing number of cancer survivors. Recent estimates indicate that there are nearly 14 million cancer survivors in the US, more than the over 7 million cancer survivors reported in 1992 [1–3]. Although research examining sociodemographic and healthcare characteristics of nationally representative samples of cancer survivors has been conducted [4, 5], differences in these characteristics over time have rarely been examined in population-based studies.

In 1992, the National Health Interview Survey (NHIS) examined characteristics of cancer survivors as part of its Cancer Control Supplement for the first time. Hewitt and colleagues published findings from the 1992 NHIS about numerous self-reported cancer survivor characteristics, including demographic information, cancer type, frequency of second opinion concerning type of cancer treatment, counseling and support group services, patient education, contact with cancer organizations, participation in clinical trials, health and life insurance coverage, and concerns with employment [4]. Findings showed that cancer survivors were largely females, over the age of 65 years, and were most often diagnosed with female gynecologic, breast, colorectal, and prostate/male reproductive cancers [4]. Additionally, 44% of cancer survivors reported not receiving a second opinion about their cancer treatment and very few survivors reported participating in clinical trials or receiving counseling/supportive services [4]. Lastly, one in nine survivors reported being denied health or life insurance coverage due to their cancer diagnosis and one in five survivors reported employment concerns [4]. As a result of this and an emerging body of studies [6, 7] illustrating gaps in the followup care of and health disparities experienced by survivors, the Institute of Medicine (IOM) published a report highlighting the importance of tracking and providing quality care and support for survivors' medical, psychosocial, employment, and insurance coverage needs, while also emphasizing the need for additional research and clinical trials [8, 9]. In 2008, the IOM released an additional report, “Cancer Care for the Whole Patient”, describing the psychosocial needs of cancer survivors including information and emotional support about diagnosis and treatment, resources for transportation and financial concerns, and issues related to disruptions in employment or academic progression, while providing recommendations about supportive and psychosocial health services to address these concerns [9].

In 2010, NHIS again collected data on cancer survivors in order to examine demographic, diagnostic, treatment, and other healthcare characteristics of this growing population. In response to IOM and other reports advising tracking quality care and characteristic of survivors overtime, our study compares reported demographics, cancer diagnosis and treatment, psychosocial care, research participation, and insurance coverage between cancer survivors who responded to the 1992 NHIS and those who responded to the 2010 NHIS. 

## 2. Methods

### 2.1. Data Sources

The NHIS is a population-based, cross-sectional household survey conducted annually by the Centers for Disease Control and Prevention (CDC), National Center for Health Statistics (NCHS), and administered by the US Census Bureau. Since 1957, the NHIS has collected data on civilian, noninstitutionalized US adults (aged 18 years or older) in order to examine healthcare status, access, and progress made towards improving health outcomes in the United States. Beginning in 1987, the NHIS began collecting data on cancer control and epidemiology. Over time, the NHIS has included various survey components. The 1992 NHIS survey consisted of a two-part questionnaire. The first part, or Core Section, consisted of a set of general health and demographic questions, while the second part, or supplement sections, contained more specific questions about health topics of interest. The 1992 NHIS survey incorporated two cancer supplements: the Cancer Control Supplement (CCS) and the Cancer Epidemiology Supplement (CES), both of which included questions on cancer survivorship (Cancer Survivorship Section). 

These questions were administered, using a split survey design, to one randomly selected adult aged 18 years and older in each of the 49,401 households sampled. The administration of the Cancer Survivorship Section on each supplement was suspended during the third quarter of the field administration due to budgetary constraints, yielding an overall response rate of 87% for the CCS and an 86% response rate for the CES [4]. Starting in 2000, cancer control data (which primarily focus on risk factors and screening behavior) have been collected in one CCS every five years. Additionally, all NHIS surveys administered after 1997 were revised to include additional questions on insurance coverage, healthcare access, and health behaviors. The 2010 administration of the NHIS was the first survey, since 1992, to include a more detailed cancer survivorship section within the CCS, an activity cofunded by the Division of Cancer Prevention and Control at the CDC and the Division of Cancer Control and Population Sciences at the National Cancer Institute (NCI). 

### 2.2. Measures

Similar to the 1992 NHIS survey, the 2010 NHIS administration was divided into two parts: the core and supplement sections. The CCS was fielded on the 2010 NHIS survey; however, several questions were revised since the 1992 administration. Topic areas covered in the current analysis and examined in both 1992 and 2010 included demographic characteristics (age, gender, race/ethnicity, marital status, education, and employment status and region), cancer history (number of cancers, cancer type, age at diagnosis and years since diagnosis, cancer treatments, and participation in cancer research studies or clinical trials), and healthcare characteristics (impact of cancer on insurance coverage and counseling and supportive group participation related to cancer). Demographic (age, race/ethnicity, marital status, education, and employment status) and cancer history (number of cancers, cancer type, age at diagnosis, and years since diagnosis) data were recoded for both 1992 and 2010 surveys. Reported age at the time of interview and at the time of diagnosis was recoded to reflect age distributions that are widely used age parameters for adolescents/young adults, middle adulthood, and older adulthood (18–29; 30–44; 45–54; 55–64; 65 and older). While data on racial and ethnic groups other than Non-Hispanic White, Non-Hispanic Black, and Hispanic were collected during survey administration, the sample sizes for these groups were too small for interpretation. Notably, data concerning cancer type was based on Surveillance Epidemiology and End Results (SEER) recodes of definitions for cancer sites from the International Classification of Diseases for Oncology, 3rd edition (ICD-O-3) [10]. In the 1992 NHIS survey, data on healthcare characteristics regarding treatment type, participation in research studies/clinical trials, impact of cancer on insurance provision, and involvement and interest in counseling or support groups were only collected for cancer survivors who were less than 10 years after diagnosis. Data on healthcare characteristics for respondents to the 2010 NHIS survey were therefore analyzed separately for survivors who were less than 10 years after diagnosis and those 10 years or more after diagnosis in order to create a more homogeneous comparison sample. 

 For the purposes of the current analyses, we included all respondents who reported ever having been diagnosed with cancer (cancer survivors). Nonmelanoma skin cancers were excluded in both samples (1992 and 2010) as they are rarely invasive and are nearly always treated in a doctor's office with no followup. In 1992, there were a total of 24,040 respondents to the NHIS, of whom 1553 (6.4%) reported ever having cancer. A total of 1018 (4.2%) respondents were included in the current analyses, excluding those who reported nonmelanoma skin cancers, skin cancers of unknown type, and unknown or ill-defined site (*n* = 535). In the 2010 NHIS, there were a total of 27,157 respondents, 2333 (8.5%) of whom reported ever having cancer. We excluded 615 respondents who reported their most recent cancer was for a noneligible site (nonmelanoma skin or other skin). After exclusions, a total of 1718 (6.3%) respondents from the 2010 NHIS were included in the current analyses. 

### 2.3. Statistical Analysis

We used descriptive statistics to examine differences in reported demographic, cancer history, and healthcare characteristics among cancer survivors who were respondents to the 1992 NHIS compared to those responding to the 2010 NHIS survey. In order to evaluate whether trends over time were unique to cancer survivors, additional descriptive analyses examining changes in demographics between the 1992 and 2010 NHIS surveys among respondents with no reported history of cancer were also conducted. In our analyses, we obtained parameter estimates by maximum likelihood techniques. To account for the complex survey design, SAS (SAS Institute, Cary, NC) version 9.2 and SAS-callable SUDAAN release 10 (Research Triangle Institute, Research Triangle Park, NC) were used to conduct all data analyses. Data were suppressed and considered statistically unreliable when the denominator was based on <50 sample cases or the Relative Standard Error (RSE) of the estimate was >30 percent [11]. Where data items were reported consistently in the 1992 and 2010 NHIS, chi square tests were used to test for significance differences (*P* < 0.05). Data items that were not reportedly consistently from 1992 to 2010 NHIS were race/ethnicity, marital status, education, employment status, and cancer type. 

## 3. Results

The proportion of respondents who reported having a history of cancer (excluding nonmelanoma and noneligible site cancers) increased from 1992 to 2010 (4.2% versus 6.3%). In both years, most survivors were females, Non-Hispanic White, and not employed or in the labor force (Table 1).

### 3.1. Changes in Cancer Survivors' Sociodemographic Characteristics (Table 1)

The age distributions of cancer survivors responding to the NHIS survey in 1992 and 2010 were significantly different (*P* < 0.001). The proportion of survivors who reported being 30 to 44 years of age at the time of interview in 1992 was significantly more than that reported in the 2010 NHIS (16.5% (95% CI = 14.2–19.1) versus 9.4% (95% CI = 8.1–10.9)) and the proportions of those 55 to 64 and ≥65 increased (17.9% versus 22.6% and 46.9% versus 50.1%, resp.). Similar changes in age distribution were reported in the general population of survey respondents with no reported history of cancer who were 30 to 44 years of age (34.5% versus 26.9%, resp.), 55 to 64 years of age (10.9% versus 15.5%, resp.), and ≥65 years of age (14.5% versus 17.7%, resp.). The proportion of male cancer survivors rose and that of female cancer survivors fell in 2010, compared to 1992 (*P* < 0.001). Comparatively, no significant changes from 1992 to 2010 in the portion of males (48.2% versus 45.8%, *P* = 0.32, resp.) and females (51.8% versus 54.2%, *P* = 0.32, resp.) were found among respondents with no history of cancer. Fewer survivors reported being married in 2010 compared to 1992 (44.6% (95% CI = 42.1–47.2) versus 65% (95% CI = 61.9–68.0)), while no changes in employment rates and region of residence were observed. Consistent with cancer survivors, fewer respondents with no history of cancer also reported being married in 2010 (44.3%), compared to 1992 (64.6%). The most pronounced demographic difference between cancer survivors in 1992 and those in 2010 was the number of years of education completed. While the largest group of survivors reported having had no more than a high school education or GED in both 1992 (65.8% (95% CI =  62.1–69.2)) and 2010 (43.4% (95% CI = 40.9–45.9)), over twice as many survivors reported having associates, bachelor's, or graduate degrees in 2010 compared to survivors in 1992 (37.2% (95% CI = 34.5–40.0) versus 15.9% (95% CI =  13.6–18.7)). This increase of reported associates, bachelor's, or graduate degrees from 1992 to 2010 among survivors was also found among respondents without a history of cancer (20.7% versus 39%). 

### 3.2. Differences in Cancer Diagnostic Characteristics among Cancer Survivors (Table 2)

The vast majority of cancer survivors in both 1992 and 2010 reported only having one type of cancer (86.6% (95% CI = 83.9–88.9) and 89.4% (95% CI = 87.8–90.8), resp.). There was a slight drop in reported rates of two or more cancer types among survivors from 1992 to 2010 (13.4% (95% CI = 11.1–16.1) versus 10.6% (95% CI = 9.2–12.2), resp.; *P* = 0.05). Breast cancer constituted the largest group of survivors in both 1992 and 2010. Half as many survivors in 2010 reported being diagnosed with other female gynecological cancers (7.3% (95% CI = 5.9–9.1) versus 14.6% (95% CI = 12.3–17.3)) compared to respondents in 1992, while the proportion of prostate cancer survivors increased in 2010. 

A drop in the proportion of survivors between the ages of 30 and 44 years at the time of their cancer diagnosis was observed from 1992 to 2010; however, an increase in survivors reporting having a cancer diagnosis between 55 and 64 years of age was found. Additionally, we found changes in years since cancer diagnosis, with a larger percentage of survivors in 2010 reporting that they were 10 to 14 years after diagnosis or over 20 years after diagnosis (Table 2). Compared to 1992 survivors, significantly fewer survivors in 2010 reported being 1 to 4 years after diagnosis (40.8% (95% CI = 37.4–44.4) versus 29.8% (95% CI = 27.4–32.3)). 

### 3.3. Type of Treatment (Table 3)

Differences were observed regarding types of treatments reported by survivors within 10 years of diagnosis in 1992 compared to survivors in 2010 (Table 3). Over 20% of survivors in 1992 and 2010 reported receiving a chemotherapy regimen. Radiation therapy was the second most widely used treatment (after surgery) among survivors in 1992 and 2010. In 2010, survivors who were 10 years or more after diagnosis reported receiving radiation treatment less frequently than survivors within 10 years of diagnosis. The majority of cancer survivors, independent of NHIS survey administration year or the number of years after diagnosis, reported having had surgery to treat their cancer (Table 3).

### 3.4. Changes in Health or Life Insurance Coverage due to Cancer (Table 3)

In 2010, cancer survivors less than 10 years after diagnosis were significantly less likely to have health or life insurance coverage denied due to their cancer diagnosis compared to 1992 survey respondents who were less than 10 years after diagnosis, (*P* < 0.001). 

### 3.5. Participation in Clinical Trials (Table 3)

While the sample size for survivors (within <10 years of diagnosis in 1992 and 2010 survivors >10 years after diagnosis) who reported participation in clinical trials was too small for accurate interpretation, only 10.4% (95% CI = 8.5–12.8) of survivors in 2010 who were less than 10 years after diagnosis participated in clinical trials.

### 3.6. Receipt of Counseling or Participation in Support Groups (Table 3)

Approximately 85% of survivors in 1992 and 2010 reportedly did not receive counseling or participate in a support group, with the majority reporting they thought they did not need this service. While more than twice as many survivors in 2010, compared to those in 1992, reported that they did not participate in counseling or a support group because they “did not want it” (24.5% (95% CI = 21.3–28.0) versus 11.6% (95% CI = 8.4–15.8)), more survivors in 2010, particularly among those 10 years or more beyond diagnosis, reported that they “did not know” counseling and support group services were available to them (13.6% for <10 year survivors (95% CI = 11.2–16.5) and 20.9% for >10 year survivors (95% CI = 17.4–24.9), resp., versus 9.6% (95% CI = 6.9–13.1)). Among 2010 survivors diagnosed within the prior 10 years who reported that they did not participate in counseling or support groups, 43% reported that they would have been interested in receiving these services.

## 4. Discussion 

According to our results, the proportion of cancer survivors has increased over time, which is consistent with data from the Behavioral Risk Factor Surveillance Survey (BRFSS) [5] and NCI's Surveillance, Epidemiology, and End Results (SEER) [2, 3]. This growth is generally attributed to an aging population and longer survival due to implementation of effective screening tests, earlier stage at diagnosis, and better treatments [12].

 The increase from 1992 to 2010 in the proportion of survivors who were men was not found in the general population of respondents with no history of cancer. While exact reasons for the gender difference among survivors are unknown, it is possible that this is a result of the trend toward earlier diagnosis and possible over diagnosis of prostate cancer [13, 14]. Furthermore, the increasing proportion of survivors who are Non-Hispanic Black may be attributed to findings showing a continued increase in cancer survival rates of African Americans in the United States [2, 15]. However, changes in the proportion of Non-Hispanic Black survivors in the US may vary based on cancer type, stage of cancer, and other characteristics [16]. The increases in the proportion of survivors who were unmarried and with post-high-school education are consistent with overall demographic trends in the US [17]. 

 The majority of cancer survivors in both 1992 and 2010 reported only having one type of cancer in their lifetime and breast cancer was most frequently the primary cancer type. Twenty-three percent of female cancer survivors in 2010 reported being breast cancer survivors, which is consistent with SEER estimates reported in 2008 [2, 3]. However, these 2010 NHIS estimates are likely underreported given the estimated increase in overall cancer survivors after 2008 [2]. While the increase in prostate cancers reported could be the result of the rapid uptake of prostate cancer testing and subsequent diagnosis among men, these findings may be a result of the older age of the study sample. The sizable proportion of melanoma skin cancers reported in 2010 can be attributed to several factors, some of which include increases in early detection and changes in behavior patterns related to lifestyle choices, tanning, and sun exposure [18]. A decline in other reported female gynecologic cancers was also found, in part due to the decrease in the number of ovarian and uterine cancers reported by survivors aged 30–44 years. 

Differences in the age at diagnosis between 1992 and 2010 were also evident. While there was an overall decline in proportion of cancer survivors under the age of 45 years at the time of diagnosis in 2010, the most pronounced decrease was among respondents aged 30 to 44 years. This reduction was largely a result of a significant decrease in other female gynecologic cancers in this age group (1992 NHIS = 26.0%; 2010 NHIS = 10.4%). Among those 45 years or older, an increase in cancer survivors was noted in the 2010 NHIS, with the largest increase being among adults aged 55–64 years at the time of diagnosis. This increase in 2010, compared to 1992, was largely due to substantially more reported prostate (22.3% versus 7.9%, resp.; data not shown) cancers diagnosed from ages 55–64 years. 

While there are several factors affecting long-term survival for individuals diagnosed with cancer, early detection and advances in treatment and cancer related healthcare have been associated with long-term cancer survival [3, 19], and several of these advances have been reported since the 1992 survey. In accordance with the trend toward increased cancer survival, the 2010 NHIS survey yielded an increased proportion of survivors living 5 or more years after diagnosis, along with an increase in those surviving 20 years or more. While the proportion of cancer survivors living more than 5 years continues to increase, these cancer survival improvements may not be equally shared by all due to differences in timing of diagnosis, access to treatment and care, and other disparities related to sociodemographic background [20]. 

 According to the 1992 and 2010 NHIS surveys, use of surgical treatment was reported more than any other treatment by cancer survivors (Table 3). This finding was expected given that surgery is the oldest form of cancer treatment, is used for curative intent, and provides a large benefit leading to cure for breast cancer survivors [21] who made up the largest group of NHIS survivor respondents. Radiation was the second most widely used treatment among 1992 and 2010 survivors who were less than 10 years after diagnosis, which was expected given that radiation after surgery (e.g., breast conservation) is indicated for some cancers and can be helpful in reducing risk of relapse or as a curative approach for some prostate cancers [21, 22]. 

Clinical trials and research studies are needed for advances in treatment and quality care for cancer survivors. While the number of survivors in 1992 reporting involvement in clinical trials and research studies was too small for interpretation, previous researches looking at clinical trial and research study involvement in 1992 indicate that only 5% of survivors participated in such activities [4]. Compared with this previous finding, current analyses suggest a potential increase in these numbers and prevalence from 1992 to 2010. This may reflect broader public awareness about the importance of research participation and more effective [23] media campaigns for clinical trials [24]. While rates of participation in research may be increasing, overall participation is still low (9%) and maybe even lower for certain racial/ethnic groups who, historically, have been more reticent to participate in experimental research [20].

Our finding that fewer cancer survivors in 2010 reported being denied health or life insurance coverage is encouraging. Cancer survivors have traditionally reported difficulties getting approval for health and life insurance coverage due to preexisting condition clauses [4, 25]. While improvements in insurance coverage for working cancer survivors have been achieved since the implementation of the 1996 Health Insurance Portability and Accountability Act (HIPAA), a minority of survivors may still experience difficulties in achieving insurance coverage due to employment status, age, health status, income, high premium costs, and a plethora of other concerns [25]. 

No significant changes from 1992 to 2010 were reported regarding participation in counseling or support groups among cancer survivors. Only 14% of cancer survivors in both 1992 and 2010 reported participation in counseling or support groups. But, consistent with other reports of low participation in these services [5, 26], this lack of change over time is, nevertheless, surprising given the broader availability of and access to insurance coverage for psychosocial care in the past decade. These results are also concerning given that cancer survivors often experience psychosocial, neurocognitive, vocational, financial, and other related concerns across the cancer continuum [27–30]. Among 1992 and 2010 cancer survivors who did not report receiving counseling or supportive services, the majority continue to state that they “did not think they needed” these services, which may be appropriate given that many survivors report resilience after cancer [30, 31]. The moderately high numbers of cancer survivors in both study years reporting that they “did not know” these services were available are consistent with previous research reporting that primary care providers do not routinely or effectively communicate with cancer survivors about quality of life, psychosocial concerns, and supportive care [32]. It is, however, surprising that there is still a high proportion of survivors unaware of psychosocial services given recent prominent publications on these services [32, 33], the growing number of cancer outreach groups, and the increasing amount of information available on the Internet. One possible explanation for the static rates of participation in professional counseling and support groups may be the availability of support in online formats [34]. Participation in counseling or supportive service might also vary based on demographic factors (e.g., age, race/ethnicity, income, and education), health characteristics (e.g., years since diagnosis, stage of cancer, and projected survival), and the amount of support a cancer survivor might receive from their family, friends, and members of their community [34, 35]. Additionally, psychosocial services may not be readily available to cancer survivors who were not treated at large cancer centers or hospitals where psychosocial care is more likely integrated into the treatment and followup care process. 

### 4.1. Strengths, Limitations, and Future Considerations

Our study makes an important contribution to the current literature by comparing estimates of healthcare characteristics for cancer survivors responding to the NHIS survey before and after the release of several notable reports [4, 8], which illustrated gaps and needs in cancer care and survivorship. The results of our study, which were based on data from a large sample of US adults, are useful in assessing changes in cancer characteristics, healthcare provision, involvement in clinical trials and supportive services (e.g., counseling), and changes in sociodemographic characteristics of cancer survivors over time. While our findings show improvements in insurance coverage, gaps in psychosocial care illuminated in the 2005 IOM publication [8] are still evident. Our use of population-based self-report data also complements other data sources (e.g., SEER) by allowing examination of important aspects of survivorship not captured by registries.

Limitations to our study include possible recall and differential misclassification biases, as well as the effects of differences in how respondents interpreted survey questions. Respondents who were very ill may have been less likely to participate in the survey. Further, these results are not generalizable to institutionalized individuals because they are not included in the NHIS sample frame. The NHIS data are adjusted to the civilian noninstitutionalized population through poststratification and weighting. While the sample of US adults surveyed was large, the smaller size of the subgroup of cancer survivors limits statistical power. Another limitation is possible self-reported misclassification of cancer type that would increase or reduce reported rates [36]. Additionally, changes made to survey questions over time limited comparability among questions, and small numbers precluded interpretation of estimates for some questions. Lastly, given the large number of statistical comparisons, some findings may be spurious.

The characteristics of cancer survivors are changing over time, and understanding these changes is a first step in meeting the needs of this growing population. Fortunately, survivors are living longer after diagnosis, and the negative impact of cancer on insurance coverage has declined. However, opportunities exist to improve supportive services to survivors and to increase participation in clinical trials. We suggest that future studies of cancer survivors continue to examine the impact that demographic, as well as cancer and healthcare, characteristics play on survivorship outcomes, especially in the context of anticipated changes brought on healthcare legislation that are likely to affect the ways in which service delivery, insurance coverage, and overall healthcare are employed over time [37]. Additional research should also examine access to cancer clinical trials, counseling, and supportive services, as well as factors affecting participation in these activities and ways of improving awareness and utilization of these services.

## Figures and Tables

**Table 1:** Demographic characteristics of cancer survivors, 1992 and 2010 NHIS.

Characteristic	1992 cancer survivors		2010 cancer survivors		*P* value
	*N*	% of total (95% confidence interval)	*N*	% of total (95% confidence interval)	
Total	1018		1718		
Age at interview (years)					
18–29	59	5.9 (4.4–7.8)	58	3.6 (2.7–4.7)	<0.001
30–44	169	16.5 (14.2–19.1)	171	9.4 (8.1–10.9)	
45–54	118	12.9 (10.5–15.7)	261	14.3 (12.7–16.1)	
55–64	173	17.9 (15.3–20.7)	392	22.6 (20.5–24.8)	
65+	499	46.9 (43.3–50.5)	836	50.1 (47.5–52.7)	
Gender					
Male	278	31.4 (28.1–35.0)	625	36.6 (34.2–39.1)	<0.001
Female	740	68.6 (64.9–71.9)	1093	63.4 (60.9–65.8)	
Race/ethnicity					
Non-Hispanic, White	867	88.6 (86.2–90.6)	1239	82.4 (80.6–84.0)	†
Non-Hispanic, Black	84	6.5 (5.1–8.4)	245	9.1 (7.9–10.5)	
Hispanic	∗	∗	167	5.8 (4.9–6.9)	
Other	∗	∗	67	2.7 (2.0–3.6)	
Marital status					
Married	510	65. 0 (61.9–68.0)	766	44.6 (42.1–47.2)	†
Unmarried	505	35.0 (32.0–38.1)	951	55.4 (52.8–57.9)	
Education					
<High school/high school/GED	673	65.8 (62.1–69.2)	768	43.4 (40.9–45.9)	†
Some college	186	18.3 (15.6–21.4)	321	19.4 (17.3–21.8)	
Associate degree/college grad/more	156	15.9 (13.6–18.7)	619	37.2 (34.5–40.0)	
Employment status					
Employed	359	36.6 (33.4–40.0)	576	34.1 (31.8–36.5)	†
Not employed or not in labor force (18+)	659	63.4 (60.0–66.6)	1142	65.9 (63.5–68.2)	
Region					
Northeast	187	18.7 (15.5–22.4)	290	18.2 (16.0–20.7)	0.817
Midwest	279	27.4 (23.7–31.4)	428	25.8 (23.7–28.0)	
South	345	34.6 (30.6–38.8)	614	35.0 (32.6–37.5)	
West	207	19.4 (16.5–22.6)	386	20.9 (18.7–23.4)	

*Suppressed if the denominator is <50 or the relative standard error (RSE) is >30%.

Differences between 1992 and 2010 data were tested using chi-square tests.

^†^Statistical tests were not conducted where data were not reported consistently over time.

Subtotals presented do not equal actual totals because respondents with missing data for a particular characteristic are not included in distribution counts for that characteristic.

**Table 2:** Cancer diagnosis characteristics of survivors, 1992 and 2010 NHIS.

Characteristic	1992 cancer survivors		2010 cancer survivors		*P* value
	*N*	% of total (95% confidence interval)	*N*	% of total (95% confidence interval)	
Total	1018	100.00	1718	100.00	
How many types of cancer have you had					
1	878	86.6 (83.9–88.9)	1542	89.4 (87.8–90.8)	0.05
2+	140	13.4 (11.1–16.1)	176	10.6 (9.2–12.2)	
Site or cancer type					
Breast	217	20.6 (17.7–23.8)	393	22.9 (20.7–25.2)	†
Cervical	123	11.3 (9.2–13.7)	154	8.8 (7.4–10.4)	
Colorectal	104	10.2 (7.9–12.9)	140	8.0 (6.8–9.5)	
Hematologic malignancies	∗	∗	103	6.4 (5.2–7.9)	
Lung and other respiratory system	∗	∗	57	3.6 (2.7–4.7)	
Other female genital system	159	14.6 (12.3–17.3)	123	7.3 (5.9–9.1)	
Prostate	78	8.9 (7.0–11.4)	231	13.1 (11.7–14.7)	
Skin (melanoma)	∗	∗	142	9.5 (8.1–11.1)	
Urinary system	∗	∗	81	5.2 (4.0–6.6)	
Other	175	16.6 (14.3–19.2)	254	15.3 (13.5–17.2)	
Age at diagnosis of most recent cancer (years)					
0–29	154	15.2 (12.8–17.9)	221	12.9 (11.3–14.7)	0.002
30–44	235	23.9 (21.2–26.9)	318	18.2 (16.3–20.2)	
45–54	142	14.5 (12.2–17.2)	311	17.9 (16.0–19.8)	
55–64	178	18.6 (15.7–21.8)	395	22.8 (20.7–25.0)	
65+	288	27.8 (24.7–31.2)	458	28.3 (25.9–30.7)	
Years since diagnosis of most recent cancer					
<1	∗	∗	89	5.8 (4.6–7.2)	<0.001
1 to <5	388	40.8 (37.4–44.4)	479	29.8 (27.4–32.3)	
5 to <10	220	21.7 (18.9–24.8)	350	21.8 (19.6–24.3)	
10 to <15	127	12.2 (10.2–14.6)	267	16.8 (14.9–18.9)	
15 to <20	94	9.18 (7.3–11.5)	132	8.2 (6.9–9.7)	
20+	135	12.8 (10.6–15.3)	273	17.6 (15.5–19.9)	

*Suppressed if the denominator is <50 or the RSE is >30 percent.

Differences between 1992 and 2010 data were tested using chi-square tests.

^†^Statistical tests were not conducted where data were not reported consistently over time.

Subtotals presented do not equal actual totals because respondents with missing data for a particular characteristic are not included in distribution counts for that characteristic.

**Table 3:** Health care characteristics of cancer survivors, 1992 and 2010 NHIS.

Characteristic	1992 cancer survivors (dx <10 years ago)		2010 cancer survivors (dx <10 years ago)		*P* value	2010 cancer survivors (dx ≥10 years ago)	
	*N*	% of total (95% confidence interval)	*N*	% of total (95% confidence interval)		*N*	% of total (95% confidence interval)
Type of treatment for most recent cancer							
Any chemo	134	20.5 (17.1–24.3)	259	26.4 (23.6–29.4)	0.072	150	24.7 (21.5–28.1)
Any radiation	159	24.5 (21.2–28.2)	286	30.4 (27.2–33.8)	0.373	135	21.6 (18.4–25.2)
Any surgery	445	68.5 (64.1–72.6)	619	66.6 (63.3–69.7)	0.108	456	74.5 (70.1–78.5)
Other	175	26.2 (22.2–30.7)	157	17.1 (14.4–20.2)	†	95	15.1 (12.2–18.5)
Participate in a research study/clinical trials as part of cancer treatment							
Yes	∗	∗	103	10.4 (8.5–12.8)	<0.001	∗	∗
No	615	95.3 (92.8- 97.0)	829	89.6 (87.2–91.5)		564	92.4 (89.7–94.5)
Ever denied health or life insurance coverage because of cancer							
Yes	63	10.9 (8.0–14.8)	59	5.9 (4.6–7.7)	<0.001	54	8.9 (6.7–11.7)
No	580	89.1 (85.2–92.0)	878	94.1 (92.4–95.4)		558	91.1 (88.3–93.3)
After diagnosis did you receive any counseling or join support group							
Yes	96	14.4 (11.6–17.6)	147	14.1 (12.1–16.5)	0.378	84	12.6 (10.0–15.6)
No	549	85.6 (82.4–88.4)	791	85.9 (83.5–87.9)		527	87.4 (84.4–89.9)
If you did not receive counseling, what was the reason							
I did not know it was available	54	9.6 (6.9–13.1)	117	13.6 (11.2–16.5)	<0.001	109	20.9 (17.4–24.9)
I did not want it	59	11.6 (8.4–15.8)	182	24.5 (21.3–28.0)		103	19.4 (16.0–23.4)
I did not think I needed it	339	64.2 (59.1–69.0)	389	50.7 (46.7–54.5)		244	47.2 (42.7–51.8)
Other	73	14.6 (11.2–18.8)	94	11.2 (9.1–13.7)		67	12.5 (9.9–15.5)
If you did not receive counseling, would you have been interested							
Yes	∗	∗	54	42.9 (33.6–52.8)	0.058	∗	∗
No	∗	∗	59	57.1 (47.2–66.4)		62	63.2 (52.6–72.7)

*Suppressed if the denominator is <50 or the relative standard error (RSE) is >30%.

Differences between 1992 and 2010 data were tested using chi-square tests.

^†^Statistical tests were not conducted where data were not reported consistently over time.

Subtotals presented do not equal actual totals because respondents with missing data for a particular characteristic are not included in distribution counts for that characteristic.
